# Immunoglobulin a nephropathy as the first clinical presentation of Wilson disease: a case report and literature review

**DOI:** 10.1186/s12876-021-01954-8

**Published:** 2021-10-19

**Authors:** Yong-Zhe Zhang, Geng Jian, Ping He, Rui Yu, Mi Tian, Yan Wu, Bei-Ru Zhang

**Affiliations:** 1 Department of Nephrology, Shengjing Hospital of China Medical University, 36 Sanhao Street, Shenyang, 110004 China; 2 Department of Pathology, School of Basic Medica Sciences, Southern Medical University, 1838, North Guangzhou Avenue, Guangzhou, 510515 China

**Keywords:** Proteinuria, IgA nephropathy, Wilson disease, Copper metabolism, Case report

## Abstract

**Background:**

Wilson disease (WD) is a rare genetic disorder of copper metabolism. Differences in copper tissue accumulation lead to various clinical manifestations, including some atypical presentations. The complex clinical features of WD make diagnosis challenging, delaying the best chance for treatment.

**Case presentation:**

We report a case of a 26-year-old man with nephritis-range proteinuria and elevated serum creatinine. The renal pathology indicated immunoglobulin A (IgA) nephropathy and tubular injury, which was inconsistent with glomerular lesions. Cirrhosis was also detected by imaging examination. Considering both kidney injury and liver damage, WD was suspected. Based on results showing abnormal copper metabolism, corneal Kayser–Fleischer rings, and genetic disorders in the *ATP7B* gene, the patient was finally diagnosed with WD. After treatment with oral penicillamine, zinc sulfate and losartan, the patient showed alleviation of both WD and nephropathy after 3 years of follow-up. He maintained a good quality of daily life.

**Conclusion:**

This case highlights that unexplained neurological and liver symptoms in patients with IgA nephropathy can be clues for WD.

## Background

Wilson disease (WD), also known as hepatolenticular degeneration, is an autosomal recessive hereditary copper metabolic disorder disease. The prevalence of WD is 1 in 30,000, and the age of onset is between 5 and 35 years [1]. The causative pathogenic gene of WD is the *ATP7B* gene localized on chromosome 13q14.3; mutations in this gene result in reduced P-type copper transport ATPase function, leading to decreased serum ceruloplasmin synthesis and gallbladder copper-discharging dysfunction.

The most common clinical manifestations of WD include liver disease and cirrhosis, neurological disorders, and Kayser–Fleischer (K–F) rings at the corneal limbus. Diagnosis of WD is typically established based on typical clinical symptoms, signs, and examinations, particularly serum ceruloplasmin and uric copper levels, as well as mutation analysis of the *ATP7B* gene. Some patients may have a positive family history. However, as a treatable genetic disease, if patients with WD can be diagnosed in a timely and accurate manner and treated in the early stages of the disease, most will achieve a good quality of life and life expectancy similar to that of healthy individuals. However, if treatment is started at a late stage of the disease, the treatment is generally ineffective, and mortality and disability rates are relatively high. Therefore, accurate, timely identification of the disease is critical. Unfortunately, obvious symptoms of WD may be hidden behind atypical symptoms manifesting in different tissues, organs, or systems; patients with these complex and varied clinical manifestations of WD are easily misdiagnosed or neglected, leading to poor prognosis. As a less common initial presentation of WD, renal injury has been described in some case series with small samples [2].

In this report, we describe a case of WD in which foamy urine was the only clinical symptom.

## Case presentation

A 26-year-old man complained of foamy urine for 3 years without hospital examination. Proteinuria was found in routine physical examinations 1 month prior, and he attended our clinic on the basis of these results. Laboratory data were as follows: urine analysis showed proteinuria (dipstick 2 +) and hematuria (3 +), 24-h uric protein quantification was 0.75 g/day (normal range, 0–0.15 g/day), and serum creatinine (Scr) was 151 μmol/L (normal range, 88–104 μmol/L). Based on abnormalities in the examination indicators, the patient was admitted to the nephrology department with an initial diagnosis of glomerulonephritis and renal insufficiency.

Further detailed consultations and examinations were carried out. The patient felt no other discomfort. His routine physical examinations showed no obvious abnormalities. He had no edema or hypertension, and his urine output was normal. During neurological examination, the patient was found to have an imperceptible tremor in his hands. A review of medical history revealed that the patient had allergic purpura 22 years prior and pneumothorax 7 years prior. The patient denied having consumed alcohol or abused drugs and denied having a history of hepatitis or family history of chronic or genetic diseases. His parents had a nonconsanguineous marriage. Laboratory examinations indicated normal white blood cell counts and hemoglobin levels. Urinalysis showed 2 + proteinuria with microhematuria (approximately 100 erythrocytes per high-power field), and his 24-h uric protein quantity fluctuated from 0.75 to 1.1 g/day. Abnormal renal function with an Scr of 150–170 μmol/L was detected. Liver function was normal, and other laboratory examinations, including serum electrolytes, thyroid function, C-reactive protein, and serum complement (C3 and C4), were also within the normal range. Additionally, immunoglobulin levels (IgG and IgM) were normal, except for mild elevation of IgA (3.73 g/L, normal range: 0.97–3.2 g/L). Serological tests were negative for anti-nuclear, anti-neutrophil cytoplasmic, anti-glomerular basement membrane, anti-hepatitis B virus, and anti-hepatitis C virus antibodies. Routine physical examination results indicated normal electrocardiogram and color Doppler ultrasound with normal kidney size and shape. Color ultrasound of the liver, gallbladder, spleen, and pancreas indicated rough echo in the liver.

**Fig. 1:**
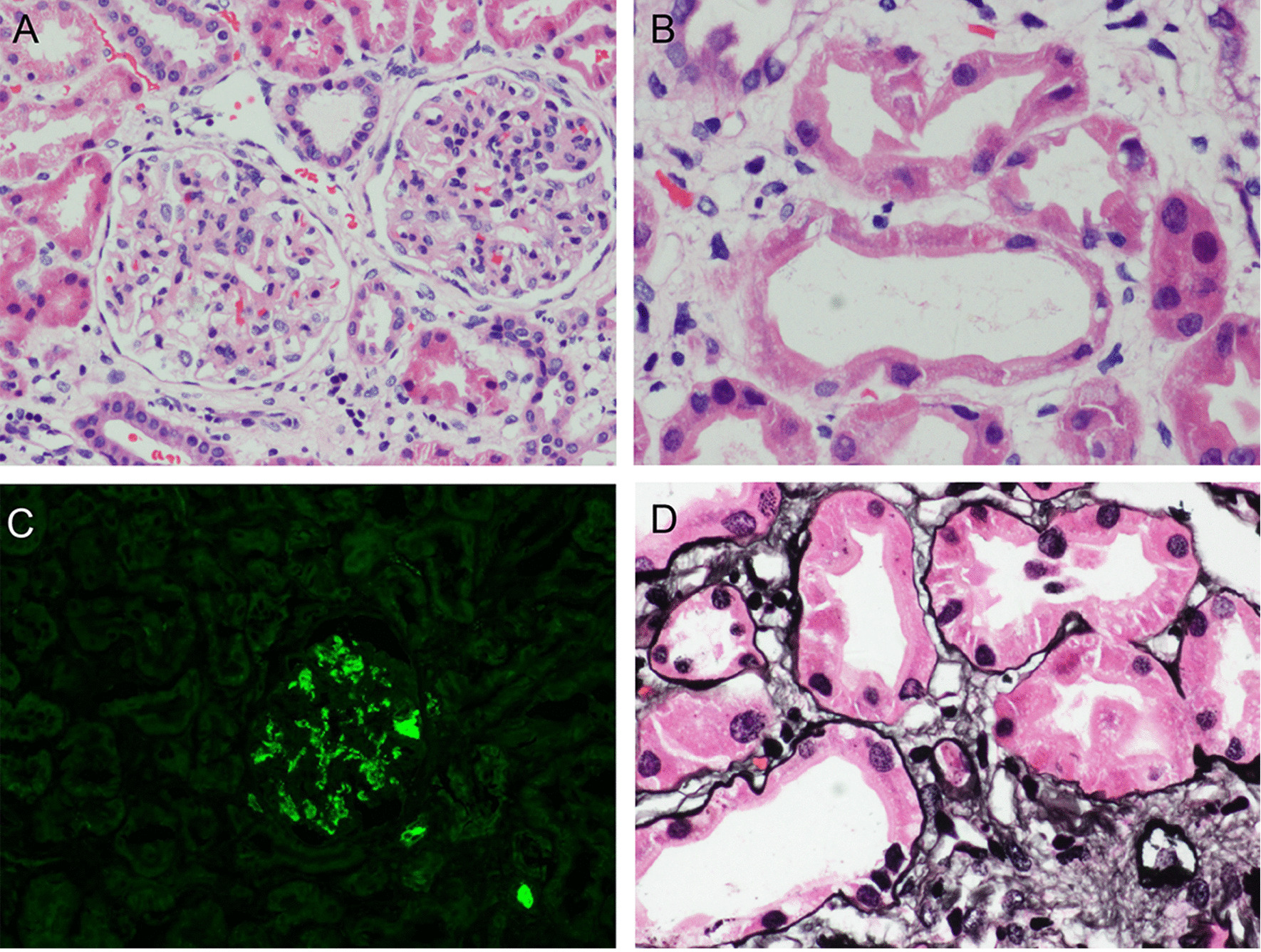
**a** Light microscopy of the kidneys showing mesangial cells and matrix proliferation in the glomeruli (H&E stain, 400 ×). **b** Light microscopy showing the disappearance of brush margins and the dilation of lumen in partial renal tubules. Renal interstitial edema and inflammatory cell infiltration were also observed (H&E, 400 ×). **c** Immunofluorescence staining of the kidneys showing bright granular deposition of IgA (400 ×). **d** Silver staining of the kidneys showing shedding of tubule bristles and enhanced interstitial edema (400 ×)

Renal biopsy was performed to identify kidney disease. Analysis of renal biopsy specimens using light microscopy showed mesangial cells and matrix proliferation with glomeruli focal segmental hyperplasia and sclerosis (1/10 glomerulus; Fig. 1a). Epithelial cells were vacuolated and showed granular degeneration. Additionally, brush margins disappeared, the lumen dilated, and focal atrophy (atrophy area of approximately 15%) was observed in some renal tubules. Interstitial focal inflammatory cell infiltration was accompanied by fibrosis, and the walls of arterioles showed no obvious pathological changes (Fig. 1b). Immunofluorescence staining showed granular deposition of IgA+++ in the mesangium (Fig. 1c). No glomeruli were observed by electron microscopy. Silver staining showed shedding of tubule bristles and enhancement of interstitial edema (Fig. 1d). The pathologic diagnosis was focal hyperplastic IgA nephropathy accompanied by acute tubular interstitial injury (Lee grade III, Oxford grade M1E0S0T1).

Diagnosis of IgA nephropathy is relatively simple based on hematuria, proteinuria, and renal biopsy results; however, renal pathology may not yield a clear diagnosis. In our study, the observed pathological changes associated with IgA nephropathy did not explain the renal dysfunction in our patient. Additionally, the degree of tubular injury was not consistent with glomerular lesions. Thus, reasons other than IgA nephropathy may contribute to tubular injury. More importantly, the young patient in our study showed rough echo of the liver in our ultrasound findings. Although we repeatedly questioned the patient about his medical history, no common causes of liver damage, such as hepatitis or a history of drugs and alcohol, were reported by the patient. Furthermore, magnetic resonance imaging of the liver revealed liver atrophy and splenomegaly. The unexplained symptoms were confusing; however, the chief physician of our institution observed the mild involuntary fingertip tremors of the patient, and WD was then considered as a potential diagnosis based on this nonspecific neurological abnormality combined with the observed liver damage.

**Fig. 2:**
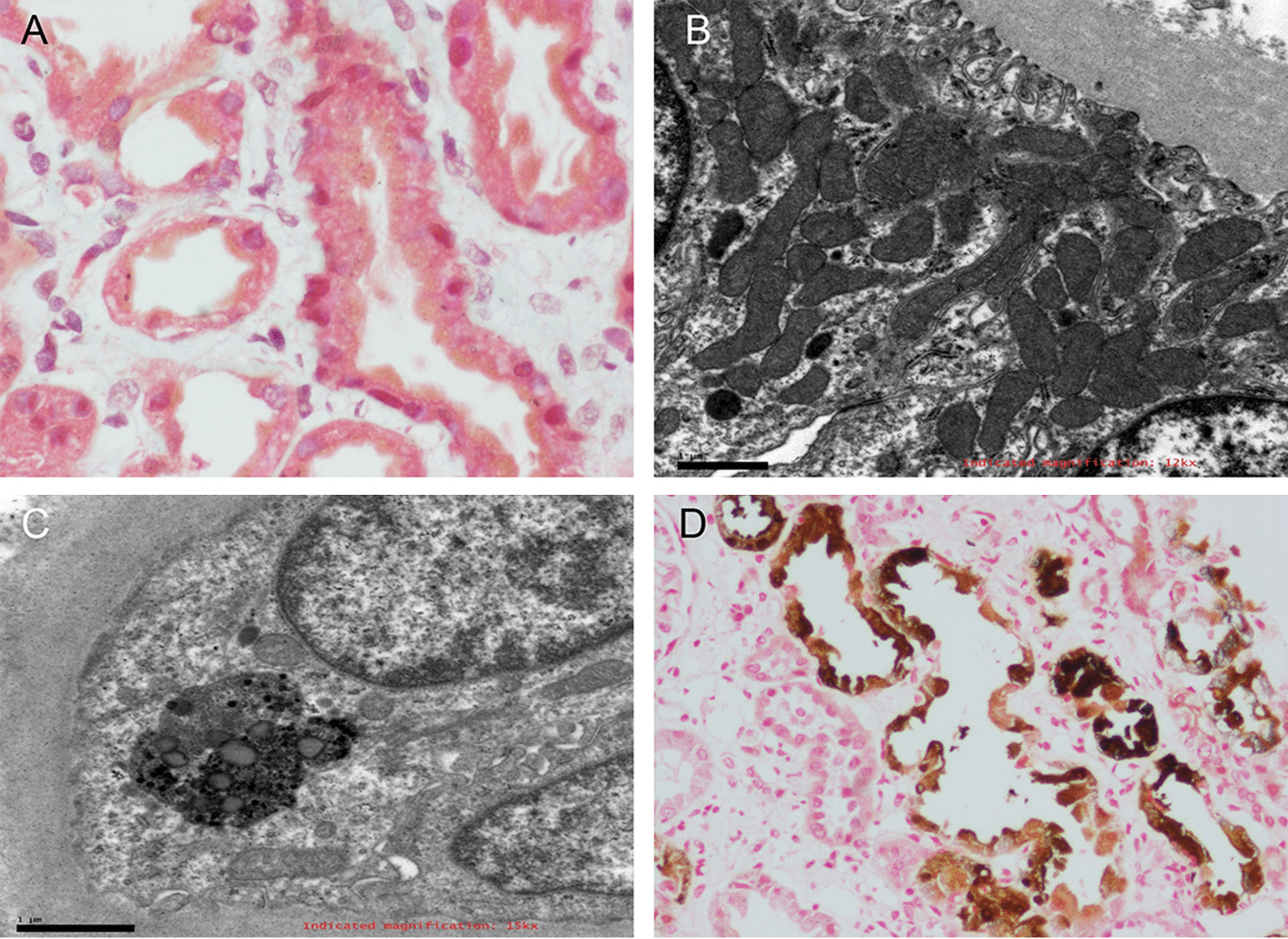
**a** Light microscopy of the kidneys showing granular deposition in the cytoplasm of renal tubules epithelial cells (H&E stain, 400 ×). **b** Some renal tubular epithelial cells showed degeneration of mitochondria in the cytoplasm. The size of mitochondria varied, the inner and outer membranes were separated, and the cristae became shorter and disappeared, as observed by electron microscopy (7500 ×). **c** Electron microscopy of some round granule deposited in lysosomes (7500 ×). **d** Brown to black deposits in renal tubular epithelial cells, as demonstrated by Timm’s copper staining (400 ×)

Renal biopsy was re-examined, and some subtle changes that had been overlooked on the first analysis were detected. For example, granular deposition in the cytoplasm of renal tubule epithelial cells was observed using light microscopy (Fig. 2a). Under electron microscopy, some renal tubular epithelial cells showed degeneration of mitochondria in the cytoplasm. The size of mitochondria varied, the inner and outer membranes were separated, and the cristae became shorter and disappeared (Fig. 2b). Moreover, lysosome size increased, and some round granules were deposited in the lysosome (Fig. 2c). Timm’s copper staining revealed some brown to black deposits in some renal tubular epithelial cells (Fig. 2d). Measurement of copper metabolism further confirmed the diagnosis of WD; lower levels of serum ceruloplasmin (0.02 g/L, normal range: 0.27–0.47 g/L) and increased urinary excretion of copper (260.4 μg/day, normal range: 10–60 g/day) were detected, although normal copper serum levels (12.52 μmol/L, normal range: 7.12–21.29 μmol/L) were also observed. The presence of K–F rings in the patient’s eyes, as observed by slit lamp examination, also supported the diagnosis of WD. To confirm this diagnosis, we also performed DNA sequence analysis and identified two mutations in the *ATP7B* gene; one was a known pathogenic mutation, whereas the other was a suspected pathogenic mutation (Table 1).

**Table 1:** Genetic analysis of the patient’s *ATP7B* gene mutations

Exon score	Location	Nucleotide mutation	Amino acid alteration	Allele	Known pathogenic
8	2333	G > T	p.Arg778Lcu	Heterozygote	Mutations
11	2621	C > T	p.Ala874Val	Heterozygote	Suspicious pathogenic mutations

Finally, the patient was diagnosed with WD. The observed kidney damage was identified as WD-associated renal injury, including renal tubulointerstitial injury and focal proliferative IgA nephropathy. The patient agreed to targeted treatment, including penicillamine 250 mg twice daily and oral zinc sulfate daily. Additionally, he was prescribed losartan to control proteinuria while monitoring renal function and blood pressure. During the 3-year follow-up, the tremor in his hands disappeared, and his 24-h uric protein level fluctuated from 0.3 to 0.5 g/day. Renal dysfunction was reversed, and Scr was maintained at approximately 110–130 μmol/L. Moreover, 24-h uric cooper dropped from 260.4 to 69 μg. The patient retained normal liver function and maintained a good quality of daily life.

## Discussion and conclusions

WD is now known to be more prevalent than once believed, particularly because of identification of *ATP7B* as a causative gene [3]. Indeed, because abnormal biliary excretion of copper owing to *ATP7B* gene mutation is a primary etiology of WD, liver abnormalities are the most common initial manifestation, and 40–70% patients diagnosed with WD exhibit liver lesions [4]. In such cases, the coexistence of asymptomatic and unexplained cirrhosis is a key feature in diagnosis. Neuropsychiatric symptoms are another common finding, detected in approximately 50% of patients [5]. In our case, we observed hand tremors in the patient, suggestive of neurological features. To date, many atypical organs showing copper deposition have been reported to be involved in WD, leading to different and complex clinical symptoms and making accurate diagnoses challenging (Table 2). Given the challenges with the diagnosis of WD, a weighted diagnostic scoring system, also known as Leipzig criteria, has been established to help clinicians evaluate patients for WD. The system encompasses many key examinations, including clinical, biochemical, and even molecular genetic testing, for making the diagnosis [6]. Although WD can be diagnosed with increased accuracy owing to our improved understanding of the disease as well as the addition of molecular diagnostic testing, diagnosis of WD is still often delayed or missed. Therefore, reports of unique cases may improve awareness of the disease.

**Table 2:** Potential clinical manifestations of WD

Target organ	Clinical features
Liver	Abnormal liver enzymes, asymptomatic hepatomegaly, acute or chronic hepatitis, cirrhosis, hepatic encephalopathy, and fulminant hepatitis [4]
Nervous system	Motor dysfunctions: dystonia, Parkinsonism, choreoathetosis, tremor, ataxia, dysarthria, oropharyngeal dysfunction; seizures [21]
	Nonmotor symptoms: school failure, personality disorders, mood changes, psychosis, cognitive abnormalities, sleep disorders, and autonomic disturbances, impulsiveness, sexual exhibitionism, inappropriate behavior [21]
Ophthalmologic manifestations	Kayser–Fleischer ring; sunflower cataract [22]; slowing of saccades, impaired upgaze, and strabismus
Blood	Hemolytic anemia (Coombs-negative hemolytic anemia) [23]; thrombocytopenia; HELLP syndrome; leukopenia
Kidney	Glomerulonephritis; nephrotic syndrome; renal tubular function disorder (renal tubular acidosis, aminoaciduria) [24]; IgA nephropathy [12]; IgM nephropathy [8]; Fanconi syndrome [25]; nephrolithiasis [26]
Musculoskeletal and joint diseases	Osteoporosis; osseomuscular; arthritis or arthralgias [27]; muscle weakness [28]
Endocrine system	Male feminization; paramenia [29]; habitual abortion [30]; infertility, sexual retardation [31]; hyperprolactinemia; hypoparathyroidism; insulinoma [32]; hypokalemia
Cardiovascular system	Electrocardiographic abnormalities; cardiac interstitial fibrosis [33], myocarditis [33]
Others	Pancreatitis [34]; cholangitis [35]; hyperpigmentation [36]; acanthosis nigricans [37]

In our current case, the patient’s earliest symptoms appeared in adulthood, and kidney abnormalities, including hematuria, proteinuria, and renal dysfunction, were the initial manifestations. Renal involvement is a relatively rare symptom of WD, particularly as part of the initial presentation of the disease. Some case reports have described cases in which renal involvement shows different manifestations, such as glomerulonephritis, nephrotic syndrome, IgA nephropathy [7], IgM nephropathy [8], and even renal function impairment [9]. However, renal tubular function disorder, which can manifest as renal tubular acidosis, aminoaciduria, and Fanconi syndrome [10], is relatively common compared with glomerular injury. A retrospective study analyzed 25 children with WD involving renal injury and showed that renal tubular injury was a relatively common symptom [11]. This result could be attributed to the increased deposition of copper in the epithelium of proximal and distal convoluted tubules, resulting in basement membrane thickening and further interference with the re-absorption function of renal tubules. In our case, relatively serious tubular injury inconsistent with glomerular lesions was observed, supporting the deposition of copper in tubular epithelial cells. IgA nephropathy was also observed in our patient, although the pathogenesis of IgA nephropathy associated with WD remains unclear. However, the IgA nephropathy in our case was more likely to be associated with WD-induced liver damage than with the direct copper deposition, because no copper deposition was found in glomeruli, consistent with another case [12]. By contrast, in the other case, the kidneys showed IgA nephropathy only, without tubular damage, and no copper deposition was detected in tubular epithelial cells. In some studies, the decreased ability of the liver to clear immunoglobulin and immune complexes has been shown to lead to increased levels in serum and deposition in the glomerulus, thereby causing nephropathy and membranoproliferative glomerulonephritis (MPGN). Gunduz reported a case of a boy diagnosed with WD in whom liver injury had resulted in cirrhosis and renal biopsy histopathology showed MPGN with deposition of IgA [13]. Similarly, a previous report described a case in Pakistan of WD complicated with IgM nephropathy [8].

Although the clinical manifestations, kidney pathology, and abnormal copper metabolism in our patient were consistent with the diagnosis of WD, investigation of gene mutations was also necessary. There are several disorders that mimic WD [14]; for example, MEDNIK syndrome, which is associated with mutations in the *AP1S1* gene, manifests as liver damage consistent with WD, neurological involvement, low serum ceruloplasmin, elevated basal 24-h urinary copper excretion, and some degree of hepatocellular copper overload [15]. In our case, there were two mutations in the *ATP7B* gene, supporting our diagnosis. To date, more than 500 known disease-associated mutations have been reported; however, no single mutation is regarded as a dominant mutation. Most patients have compound heterozygous mutations, with a different mutation on each allele of the gene [16]. Thus, different mutations in patients may contribute to the individuality of manifestations in patients with WD.

Once a diagnosis of WD is established, treatment must be initiated. d-penicillamine and zinc salts are still standard first-line treatments, and the goal of treatment is to alleviate symptoms. However, recent efforts have been made to identify new drugs to treat WD, including oral medicines (such as trientine), and increase life-long adherence [17]. Additionally, methanobactins are novel drugs that promote copper excretion [18]. Despite these advancements, new therapeutic strategies are still needed [19, 20].

In conclusion, we reported a rare case of WD with kidney disease as the first symptom. To the best of our knowledge, this is the first case in which IgA nephropathy and renal tubular injury caused by copper deposition coexisted in a patient with WD, providing insights into the characteristics and diagnosis of WD. Based on this case, we suggest that physician be aware of the potential for renal disease in patients with WD, particularly in the presence of suspicious neurological or hepatic abnormalities. Although improvements in copper metabolism monitoring methods and gene monitoring have enhanced the convenience and accuracy of WD diagnosis, the disease is still easily missed or misdiagnosed. Accordingly, case reports such as this study are expected to improve our understanding and diagnosis of WD.

## Data Availability

All available data are presented in the case.

## Abbreviations

WD: Wilson disease
IgA: Immunoglobulin A
K–F rings: Kayser–Fleischer rings
Scr: Serum creatinine
MPGN: Membranoproliferative glomerulonephritis
